# *Eumycetozoa* = *Amoebozoa*?: SSUrDNA Phylogeny of Protosteloid Slime Molds and Its Significance for the Amoebozoan Supergroup

**DOI:** 10.1371/journal.pone.0006754

**Published:** 2009-08-25

**Authors:** Lora L. Shadwick, Frederick W. Spiegel, John D. L. Shadwick, Matthew W. Brown, Jeffrey D. Silberman

**Affiliations:** Department of Biological Sciences, University of Arkansas, Fayetteville, Arkansas, United States of America; McGill University, Canada

## Abstract

Amoebae that make fruiting bodies consisting of a stalk and spores and classified as closely related to the myxogastrids have classically been placed in the taxon *Eumycetozoa*. Traditionally, there are three groups comprising *Eumycetozoa*: myxogastrids, dictyostelids, and the so-called protostelids. Dictyostelids and myxogastrids both make multicellular fruiting bodies that may contain hundreds of spores. Protostelids are those amoebae that make simple fruiting bodies consisting of a stalk and one or a few spores. Protostelid-like organisms have been suggested as the progenitors of the myxogastrids and dictyostelids, and they have been used to formulate hypotheses on the evolution of fruiting within the group. Molecular phylogenies have been published for both myxogastrids and dictyostelids, but little molecular phylogenetic work has been done on the protostelids. Here we provide phylogenetic trees based on the small subunit ribosomal RNA gene (SSU) that include 21 protostelids along with publicly available sequences from a wide variety of amoebae and other eukaryotes. SSU trees recover seven well supported clades that contain protostelids but do not appear to be specifically related to one another and are often interspersed among established groups of amoebae that have never been reported to fruit. In fact, we show that at least two taxa unambiguously belong to amoebozoan lineages where fruiting has never been reported. These analyses indicate that we can reject a monophyletic *Eumycetozoa*, *s.l.* For this reason, we will hereafter refer to those slime molds with simple fruiting as protosteloid amoebae and/or protosteloid slime molds, not as protostelids. These results add to our understanding of amoebozoan biodiversity, and demonstrate that the paradigms for understanding both nonfruiting and sporulating amoebae must be integrated. Finally, we suggest strategies for future research on protosteloid amoebae and nonfruiting amoebae, and discuss the impact of this work for taxonomists and phylogenomicists.

## Introduction

**Figure 1:**
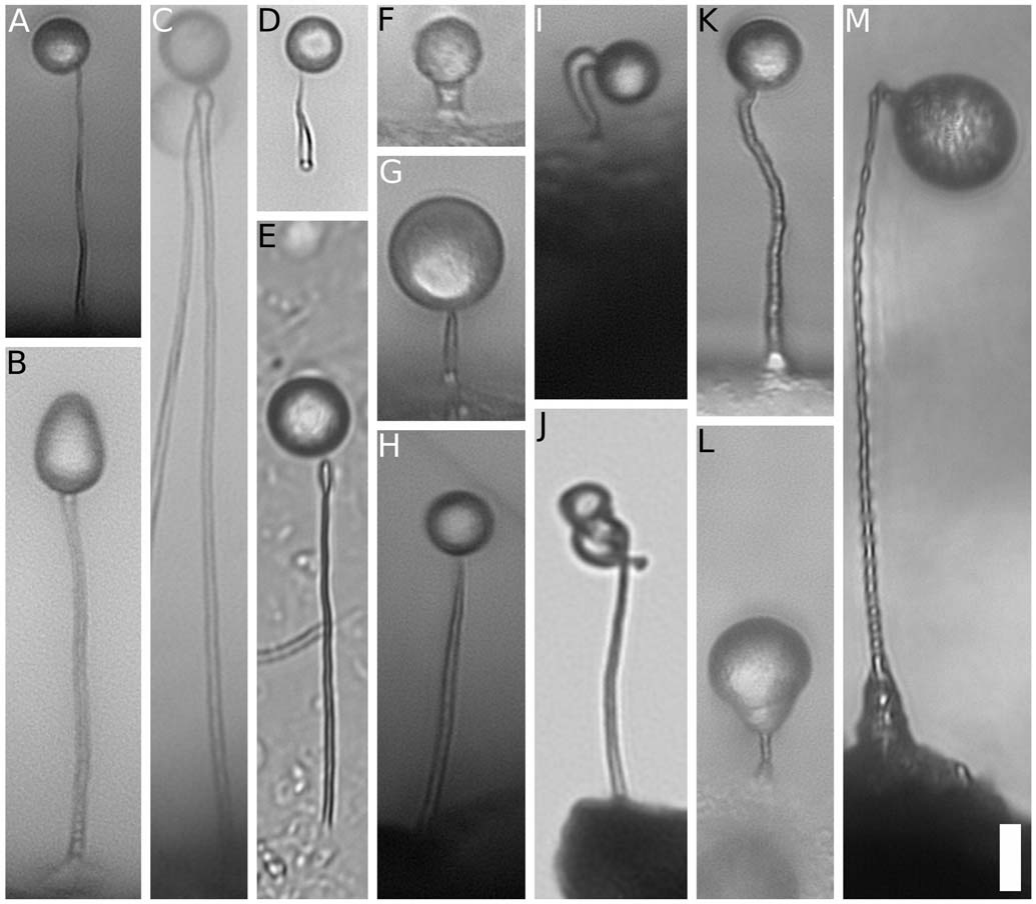
Protosteloid Fruiting Bodies. Brightfield light micrographs of standing protosteloid fruiting bodies. A) *Protostelium mycophaga*, B) *Nematostelium ovatum*, C) *Ceratiomyxella tahitiensis*, D) *Soliformovum expulsum*, E) *Soliformovum irregularis*, F) *Cavostelium apophysatum*, G) *Schizoplasmodiopsis amoeboidea*, H) *Tychosporium acutostipes*, I) *Clastostelium recurvatum*, J) *Protosporangium articulatum*, K) *Protosteliopsis fimicola*, L) isolate LHI05, M) *Endostelium zonatum*. Scale bar is 10 µm.

A microscopic drop of water resting upon the tip of a fine hair, this is the search image for organisms historically called protostelids. When researchers see this, they know they might be looking at a protostelid fruiting body. The so-called protostelids are amoebae that make simple fruiting bodies consisting of a delicate stalk that supports one or a few spores (Figure 1) [1]–[3]. Other fruiting amoebae, the dictyostelids and myxogastrids (also referred to as myxomycetes), make relatively complex fruiting bodies with many cells: the dictyostelids by aggregative fruiting and the myxogastrids by division of large, multinucleate cells into uninucleate spores [1], [4], [5]. Olive [1], [see also 2], [6] thought that the simplicity of protostelid fruiting bodies suggested that the ancestors of dictyostelids and myxogastrid amoebae might have made protostelid-like fruiting bodies. Olive called this group the taxon *Eumycetozoa* and envisioned the monophyletic taxa *Myxogastria*, and *Dictyostelia* arising from a paraphyletic taxon *Protostelia* (Figure 251 of [1]) [7]. Different interpretations of morphology by both Olive and Spiegel were used to call this idea into question [1], [2], [6], [8]. However, early molecular phylogenies that included species from all three groups suggested that there might be a clade of eukaryotes that includes myxogastrids, dictyostelids, and protostelids [8]–[10]. Was this support for the taxon *Eumycetozoa*?

Since 1654 when the first record of a myxogastrid was purported to be a fungus, the insidious perception that fruiting body formation has phylogenetic relevance has perpetuated a divide between those biologists who study amoebae and those biologists who study amoebae that fruit (for reviews see [1], [11], [12]). Fruiting amoebae are those amoebae that make spores, usually supported by stalks, at some point during their life-cycle, and are typically studied by classically trained mycologists. These amoebae are identified, isolated, and described beginning with their fruiting bodies (for reviews see [1]–[5], [11]). Amoebae that are not known to fruit are typically studied by classically trained protistologists. Such amoebae are identified, isolated, and described by their amoebal morphology and sometimes by their cysts (for review see [13], [14]). The reasons for this scientific divide are historical and methodological, not biological.

Until the last decade, when molecular phylogenies began to show otherwise, amoebae were thought of as a polyphyletic assemblage of eukaryotes. Baldauf *et al*. [10] were the first to show that some classical amoebae and some dictyostelids and myxogastrids grouped together. There has been a flush of recent molecular phylogenetic evidence showing that some fruiting and some nonfruiting amoebae belong to the supergroup *Amoebozoa* [10], [15]–[27]. Taxonomic sampling of protostelids has been a major limitation in all of these studies. In fact, those protostelids that have been included in phylogenies do not span the breadth of the purported morphological groups of protostelids [2], [8]. In fact, the ribosomal small subunit RNA gene (SSU) sequences from only two very closely related species have been used as exemplars in all the above studies that include any protostelids . We think that the term protostelid has led to confusion in the literature because it implies an evolutionarily cohesive taxonomic unit [2], [7], while at the same time, [2], [8] the term protostelid is used to describe a morphology [2]. Therefore, to avoid this double meaning we will hereafter refer to these organisms in a descriptive sense as protosteloid amoebae, not as protostelids.

**Figure 2:**
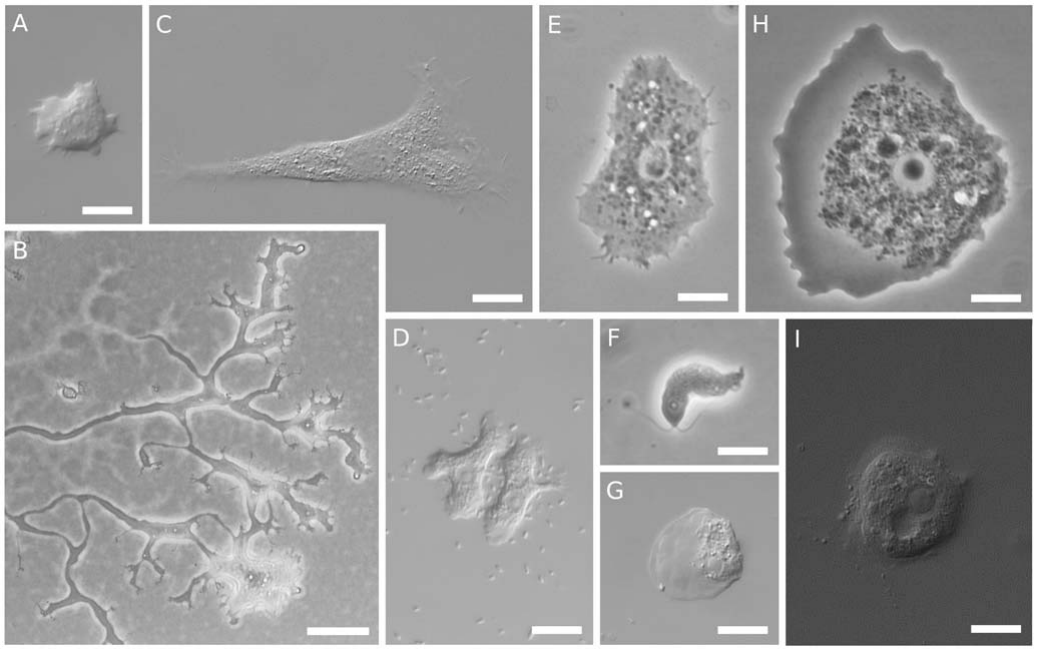
Protosteloid Amoebae. Light micrographs of protosteloid amoebae. A) *Protostelium mycophaga* differential interference contrast microscopy (DIC), B) *Nematostelium ovatum* phase contrast microscopy (PC), C) *Soliformovum expulsum* DIC, D) *Cavostelium apophysatum* DIC, E) *Schizoplasmodiopsis amoeboidea* PC, F) *Protosporangium articulatum* PC, G) *Protosteliopsis fimicola* DIC, H) isolate LHI05 PC, I) *Endostelium zonatum* DIC. Scale bars are 10 µm except B which is 50 µm.

**Figure 3:**
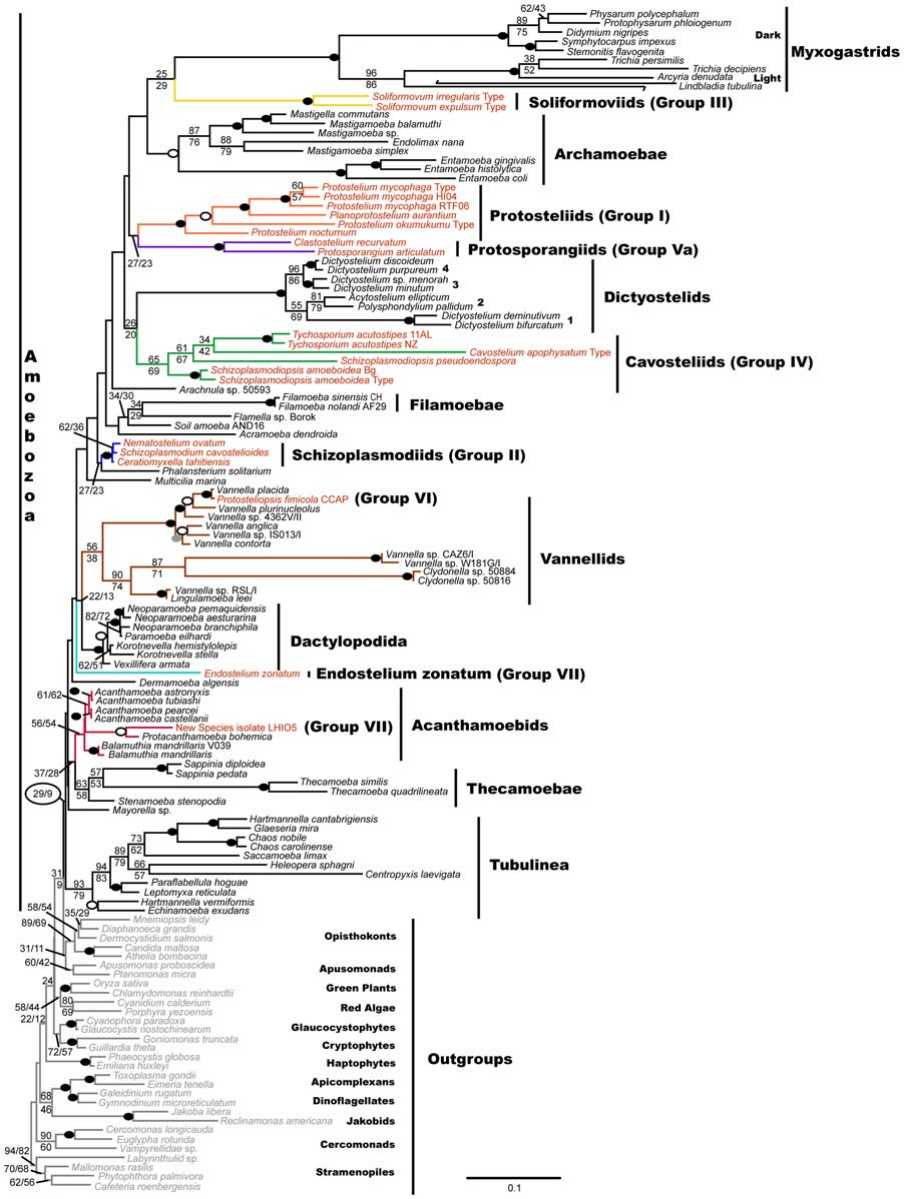
129 Taxa SSU Maximum Likelihood Tree of Protosteloid Amoebae, Other Amoebozoans and Eukaryotes as Outgroups. Colored branches indicate lineages in which protosteloid fruiting occurs. Black branches highlight amoebozoan lineages and gray branches show other eukaryotes used as outgroups. Red, black, and gray fonts indicate species of amoebae with protosteloid fruiting, nonfruiting amoebozoans, and other eukaryotes used as outgroups, respectively. To allow the figure to fit legibly on a single page, and to conserve the long branch length, the long branch leading to *Lindbladia* has been broken and shifted above and left. One hundred twenty nine taxa and 1,169 aligned positions were used to infer the optimal maximum likelihood (ML) tree in RAxML 7.0.4 using the following model (GTR + Γ, α = 0.513834, 20 discrete rate categories). ML bootstrap values from analyses of 1,000 RAxML datasets and 1,000 GARLI 0.96 datasets are shown above and below the node respectively. ML bootstrap values: black oval = 90–100, white oval with black outline = 80–90, gray oval = 70–80, unmarked<20. Black circle highlights the support values for monophyly of *Amoebozoa*. For the GARLI analyses, the following model was used (GTR + Γ + I, α = 0.71950104, 4 discrete rate categories). The scale bar represents evolutionary distance in changes per site.

Protosteloid amoebae have simple fruiting structures (Figure 1), and a range of highly diverse amoeboid trophic cells (Figure 2 and [1]–[3]). Analysis by Spiegel of amoebal morphology as well as fruiting led to five proposed, morphologically identifiable groups of protosteloid amoebae that he thought were good candidates for being closely related to myxogastrids and dictyostelids [2], [6]. These five groups include 28 of the 36 species described as protosteloid amoebae. Of the other eight species, one, *Echinostelium bisporum*, is clearly a myxogastrid [28], [29], and the rest are of doubtful affinity [2]. Only by including protosteloid amoebae that span this known diversity in analyses with an appropriately broad set of outgroups will it be possible to determine whether there is a clade that corresponds to Olive's [1] hypothesis that there is robust phylogenetic support for the taxon *Eumycetozoa*. If the *Eumycetozoa* hypothesis is correct, then fruiting amoebae should form a monophyletic (or natural) group that includes some protosteloid amoebae, the myxogastrids, and the dictyostelids to the exclusion of nonfruiting amoebae.

To gain insights into the relationships among protosteloid amoebae and where they fit among other amoebae, we have sequenced the SSU of 21 isolates representing 17 species of protosteloid amoebae including multiple representatives of each of the five “eumycetozoan” groups of Spiegel [2] and three other species, *Endostelium zonatum, Protosteliopsis fimicola*, and undescribed protosteloid isolate LHI05, whose morphologies suggest questionable affinity to the other purported eumycetozoans (Figures 1,2). These were included in phylogenetic analyses along with the SSU sequences from a broad range of amoebozoans (for recent reviews of *Amoebozoa* see [19], [23], [30]), and from a diverse assemblage of outgroup eukaryotes. Several cercozoans and stramenopiles were included, because Spiegel [2], [6], [8] had suggested members of both groups as possible close relatives to protosteloid amoebae. The SSU gene was chosen because it is the most widely sequenced among amoebozoans and because it has been used to support the phylogenies of a number of clearly monophyletic lineages within *Amoebozoa* [16], [17], [21], [23], [26], [27], [31]–[36] including dictyostelids [34] and myxogastrids [33]. We included multiple representatives from well supported amoebal lineages in our analysis to (a) look for congruence between our results and other amoebozoan phylogenies and (b) test whether any or all organisms described as protosteloid amoebae fell into a clade of amoebozoans that also included the myxogastrids and dictyostelids, *i.e. Eumycetozoa sensu* Olive [1]. Further, we wanted to know if protosteloid amoebae were indeed a grade of *Eumycetozoa sensu* Olive [1]. We show here that protosteloid amoebae are all members of the supergroup *Amoebozoa* and that there are several discrete lineages that include protosteloid species. There is no evidence for a group that corresponds to *Eumycetozoa sensu* Olive; rather, stalked fruiting is widespread among the supergroup.

## Results

The SSU rRNA genes of 21 isolates, representing 17 species of protosteloid amoebae were sequenced to assess their phylogenetic affinities (Table 1). The sequences ranged from 1,786 bp in *Schizoplasmodiopsis pseudoendospora* to 2,493 bp in *Endostelium zonatum* (Table 1). No group 1 introns were observed, and nearly all variation in length was contained within hypervariable regions of the SSU rRNA gene. Those seven isolates with especially short SSU genes<1,850 bp had some truncations in regions that are generally conserved across a diverse array of eukaryotes. Most SSU genes of protosteloid amoebae were AT rich with GC contents ranging from 38% in *Schizoplasmodiopsis amoeboidea* to 50% in isolate LHI05 (Table 1). Within-isolate sequence heterogeneity was detected in nine isolates, and was most extensive in *E. zonatum*, unnamed isolate LHI05, *Protosporangium articulatum*, and all isolates of *Protosteliopsis fimicola* (Table 1).

**Table 1:** Characteristics of Protosteloid Amoeba SSU rRNA Gene Sequences.

Group	Organism	Clone/PCR	bp	%GC	heterogeneity
I	*Protostelium mycophaga* type	PCR	1809	41.8	none
I	*Protostelium mycophaga* HI04	PCR	1819	41.7	none
I (I)	*Protostelium okumukumu* type	PCR	1813	45.2	none
I	*Protostelium nocturnum*	PCR	1800	45.9	1s
II	*Schizoplasmodium cavosteliodes*	2 clones	1937	44.6	not detected
II	*Nematostelium ovatum*	1 clone	1918	43.8	not detected
II	*Ceratiomyxella tahitiensis*	1 clone	1883	44.3	not detected
III	*Soliformovum expulsum* type	10 clones	1894	45.3	1y
III	*Soliformovum irregularis* type	1 clone	1898	45.8	not detected
IV	*Cavostelium apophysatum* type	2 clones	1794	47.9	not detected
IV	*Schizoplasmodiopsis pseudoendospora*	1 clone	1786	47.9	not detected
IV(I)	*Tychosporium acutostipes* NZ	1 clone	1835	46.4	not detected
IV(I)	*Tychosporium acutostipes* KE	1 clone	1856	46.3	not detected
IV	*Schizoplasmodiopsis amoeboidea* bg	10 clones	1876	38.4	none
IV	*Schizoplasmodiopsis amoeboidea* type	10 clones	1927	38.6	1r,1y
Va	*Protosporangium articulatum*	5 clones	2312	40.3	4r,5y,1k
Va	*Clastostelium recurvatum*	1 clone	2119	38.6	not detected
VI	*Protosteliopsis fimicola* OM05	PCR	1945	40.5	1y,1d
VI	*Protosteliopsis fimicola* Ken-A	PCR	1970	40.7	4r,6y,1k,1w,2s
VI	*Protosteliopsis fimicola* CCAP	Clone 4/PCR	1945	40.5	in PCR prod.
VI	*Protosteliopsis fimicola* CCAP	Clone 3/PCR	1945	40.1	in PCR prod.
VII	*Endostelium zonatum*	Clone 1/PCR	2493	43.4	in PCR prod.
(VII)	Unnamed LHI05	Clone 9/PCR	2254	50.1	in PCR prod.
(VII)	Unnamed LHI05	Clone 2/PCR	2253	50.2	in PCR prod.

SSU sequence length in base pairs (bp), %GC content, and within isolate sequence micro-heterogeneity. Organized by protostelid groups I-VII of Spiegel [2], parenthetical groups proposed in later papers [39], [57], or expected based on morphology. For sequence heterogeneity, ‘none’ = PCR product sequenced and no heterogeneity found, ‘not detected’ = 1–3 clones sequenced and no sequence heterogeneity detected, type and number of sites exhibiting heterogeneity explicitly noted by standard IUPAC code (s = C or G, y = C or T, r = A or G, k = G or T, w = A or T, d = A or G or T), ‘in PCR prod.’ = heterogeneity noted in PCR product, sequencing failed through regions of heterogeneity and multiple clones were sequenced individually.

**Figure 4:**
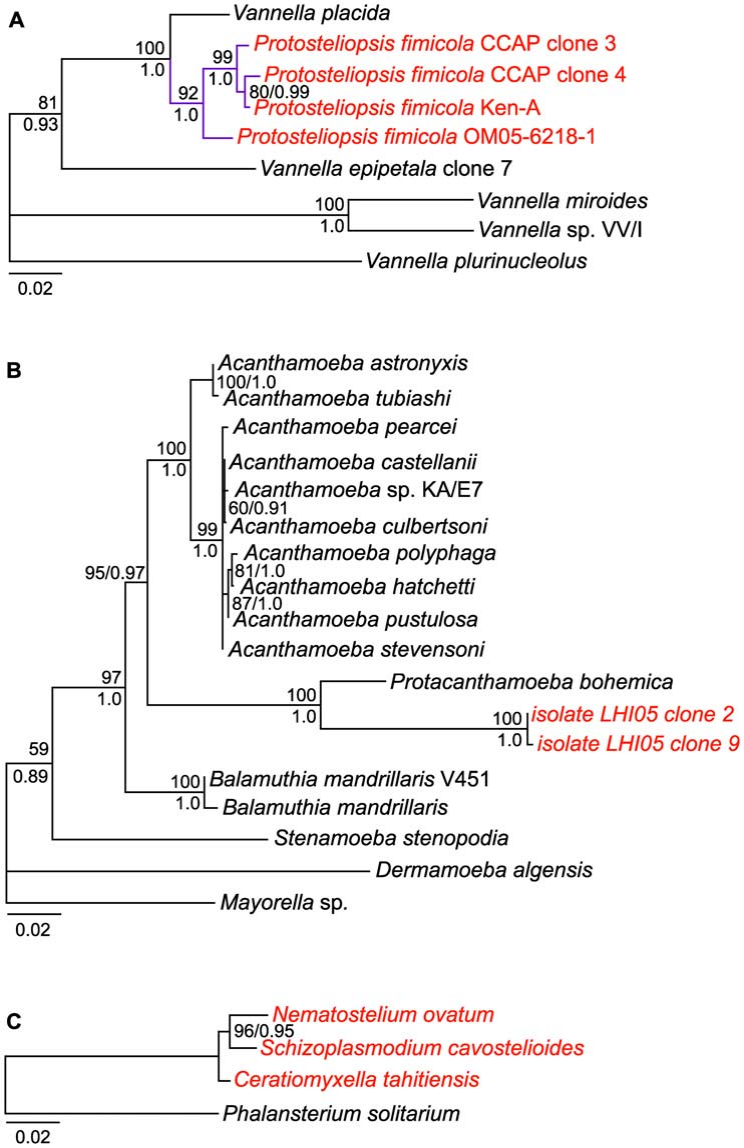
SSU Maximum Likelihood Trees Assessing Placement of Protosteloid Amoebae within Selected Clades. For all trees, the scale bars represent evolutionary distance in changes per site. Red font indicates protosteloid amoebae. ML bootstrap values from analyses of 1,000 datasets and Bayesian posterior probabilities are shown above and below the nodes respectively. A) Placement of *Protosteliopsis fimicola* among vannellids. ML tree of 9 SSU genes and 1,837 aligned positions inferred with a GTR + Γ + I (α = 0.5042, 4 discrete rate categories, and I = 0.3421) model of nucleotide substitution. For the Bayesian analyses two runs, each consisting of 4 MCMC chains, were run for 2,000,000 generations, sampling every 100^th^ tree. The first 100 trees were discarded as burnin after assessing for convergence of parameters. Purple branches highlight *Protosteliopsis fimicola* clade. B) Placement of protosteloid Isolate LHI05 among acanthamoebids. ML tree of 18 ssu genes and 1,476 aligned positions inferred with a TrN + Γ, α = 0.2228, 4 discrete rate categories model of nucleotide substitution. For the Bayesian analyses two runs each consisting of 4 MCMC chains were run for 2,000,000 generations, sampling every 100^th^ tree. The first 4,000 trees were discarded as burnin after assessing for convergence of parameters. C) Branching order of Schizoplasmodiids rooted with *Phalansterium*. Four taxa and 1,735 aligned positions were used to infer the optimal ML tree with a TrN + Γ, α = 0.3693, 4 discrete rate categories model. For the Bayesian analyses two runs each consisting of 4 MCMC chains were run for 5,000,000 generations, sampling every 100^th^ tree. The first 5,000 trees were discarded as burnin after assessing for convergence of parameters.

All of the organisms with protosteloid types of fruiting group within *Amoebozoa* in our maximum likelihood tree (Figure 3). There are several well supported clades that contain protosteloid species. Five of these clades with more than one species include protosteloid amoebae exclusively. We refer to these by the following informal designations: the **protosporangiid clade**, the **protosteliid clade**, the **soliformoviid clade**, the **cavosteliid clade**, and the **schizoplasmodiid clade**. Two species of protosteloid amoebae branch with high support within established, species rich amoebal lineages where no fruiting members have previously been reported. *Protosteliopsis fimicola* is a vannellid (Figures 1K, 2G, 3, 4A), and the undescribed isolate LHI05, is an acanthamoebid (Figures 3, 4B). So far, only one sequenced protosteloid species has no obvious close relatives, *E. zonatum* (Figures 1M, 2I, 3). We had originally included LHI05 in the analysis because its amoeba and mode of fruiting are reminiscent of *E. zonatum* (Figures 1I,M, 2H,I). However, these two taxa do not appear to be specifically related to each other. While all of the protosteloid species branched within a monophyletic *Amoebozoa* in our ML tree, the bootstrap support for a monophyletic *Amoebozoa* is lacking (Figure 3).

The monophyly of myxogastrids and the monophyly of dictyostelids are maintained in our analyses. The myxogastrids form a clade that is divided into the dark spored and light spored lineages [33], and the dictyostelids show the four clades of Schaap *et al*. [34] (Figure 3). Our highest likelihood tree has protosteloid clades as sister to the myxogastrids and dictyostelids. The soliformoviid clade is a poorly supported sister to the myxogastrids and the cavosteliid clade appears as a sister to the dictyostelids, again with weak support (Figure 3).

There is no discrete clade of *Amoebozoa* that exclusively contains all the fruiting species we included in our taxon sample in our highest likelihood tree (Figure 3). That is, we recovered no monophyletic taxon *Eumycetozoa sensu* Olive [1]. There is no clade that exclusively includes the myxogastrids, the dictyostelids, and some subset of the protosteloid species and no nonfruiting amoebae *i.e.* an exclusively fruiting clade that could be consistent with Olive's [1] *Eumycetozoa* hypothesis in a more limited sense.

There is an essentially unsupported clade that occurs in our highest likelihood tree that includes most of the protosteloid species thought to be *Eumycetozoa* by Spiegel [2]. This unsupported clade includes the protosporangiids, protosteliids, soliformoviids, cavosteliids, schizoplasmodiids, myxogastrids and dictyostelids with a number of nonfruiting amoebozoans including archamoebids, *Arachnula*, both *Filamoeba* spp., *Acramoeba*, and the amoebozoan flagellates *Multicilia* and *Phalansterium* (Figure 3).

Three clades that contain both protosteloid amoebae and amoebozoans that have never been reported to fruit were examined in more detail.

Within the poorly supported group that contains the myxogastrids, dictyostelids, many protosteloid amoebae and several nonfruiting amoebozoans, there is an interesting sister group relationship recovered between the schizoplasmodiid clade and the amoebozoan flagellate *Phalansterium solitarium*. This group appears with low support in the large tree that is restricted to 1,169 alignable postitions (Figure 3). However, *P. solitarium* is alignable with all of the schizoplasmodiids across nearly their entire SSU rRNA genes, including hypervariable regions, such that 1,735 unambiguously aligned positions are amenable to phylogenetic analyses (Figure 4C). More detailed analyses of this region of the tree using *P. solitarium* as an outgroup to the schizoplasmodiids shows that *Nematostelium ovatum* and *Schizoplasmodium cavostelioides* are sister to each other with *Ceratiomyxella tahitiensis* branching basally to them (Figure 4C).

Isolate LHI05 groups with high support within the acanthamoebids with specific and robust affinity to *Protacanthamoeba bohemica* (Figure 4B).

*Protosteliopsis* is clearly a vannellid with *Vannella placida* as its sister species (Figures 3, 4A). The sister group relationship between *Protosteliopsis fimicola* and *Vannella placida* is upheld when multiple isolates of *P. fimicola* and additional vannellids are included in a fine-scale analysis (Figure 4A).

### Hypothesis Testing

Some previous hypotheses about the relationships among the organisms traditionally considered to be eumycetozoans [1] and their relationships with other eukaryotes were not compatible with branching patterns recovered in our maximum likelihood tree. To test these hypotheses we built topologically constrained trees and compared their likelihoods to our maximum likelihood trees and bootstrap trees using the Approximately Unbiased (AU) test. Table 2 lists some important hypotheses of relationships among the purported eumycetozoans and other organisms that have been listed in the literature. Rejection of constrained trees was established at an AU test *p*-value of 0.05.

**Table 2:** *P*-Values for the AU Tests of Selected Hypotheses.

Hypothesis Tested	Constraint Tested	*p*-value	R/NR
Monophyletic protosteloid amoebae (607)	((all protosteloid amoebae) . . .);	0.037	R
*Eumycetozoa sensu lato* (606)	((all fruiting amoebozoans) . . .);	0.017	R
*Eumycetozoa sensu strictu* (608)	((I,II,III,IV,Va,D,M) . . .);	0.368	NR
*Pla* excluded from *Protostelium s.l.* (603)	((*Protostelium s.l.*)*Pla . . .*);	0.127	NR
*Ca* excluded from cavosteliids (610)	((cavosteliids)*Ca* . . .);	0.476	NR
protosporangiids sister to *Myxogastria* (436)	((Vb,M) . . .);	0.508	NR
LHI05 sister to *Ez* (605)	((LHI05,*Ez*) . . .);	0.077	NR
LHI05 excluded from acanthamoebids (604)	((acanthamoebids)LHI05 . . .);	1×10−5	R
*Pf* excluded from vannellids (602)	((vannellids)*Pf* . . .);	0.142	NR
AU test control (all taxa together) (601)	(. . .);	0.714	NR

Hypotheses are rejected at a *P-*value of <0.05. R/NR = Rejected or Not Rejected respectively. *P-*values of rejected hypotheses are shown in red. Constraints = ((constrained taxa) excluded taxa, all remaining taxa indicated by . . .);. D = dictyostelids, M = myxogastrids, I = protosteliids, II = schizoplasmodiids, III = soliformoviids, IV = cavosteliids, Va = protosporangiids, LHI05 = Isolate LHI05, *Ez* = *Endostelium zonatum*, *Pf* = *Protosteliopsis fimicola*, *Pla* = *Planoprotostelium aurantium*, *Ca* = *Cavostelium apophysatum*. See Table S2 for a comprehensive list of all 610 hypotheses tested, exact constraints used, likelihood scores, and *p-*values obtained.

Brief descriptions of some of the more interesting AU test results follow. A group that exclusively contains all the protosteloid amoebae is rejected (Table 2). *Eumycetozoa* in the strictest sense, *i.e.*, a group that includes all the protosteloid species, myxogastrids, and dictyostelids to the exclusion of other groups is also rejected (Table 2). However, *Eumycetozoa* cannot be rejected if it is defined to include only protosteliids, schizoplasmodiids, soliformoviids, cavosteliids, protosporangiids, dictyostelids, and myxogastrids, where, *E. zonatum*, *P. fimicola*, isolate LHI05, and all nonfruiting amoebozoans are excluded from the constraint (Table 2).

Within the well supported, species rich clades that contain protosteloid amoebae, *i.e*., the protosteliid clade, the cavosteliid clade, the vannellids, and the acanthamoebids, some hypotheses can be rejected and others cannot. In the protosteliid clade, while *Planoprotostelium aurantium* is nestled within the protosteliid clade in all of our highest likelihood trees, it cannot be rejected as sister to *Protostelium* (Table 2). Likewise in the cavosteliid clade, *Cavostelium apophysatum* cannot be rejected as the sister to all other cavosteliids (Table 2), nor can the protosporangiids be rejected as sister to *Myxogastria* (Table 2). Trees in which LHI05 was excluded from the acanthamoebids were soundly rejected (Table 2). *Protosteliopsis fimicola* branched as the sister taxon to the remaining vannellids when it was excluded from that group, and this was a relationship that could not be rejected (Table 2).

*Endostelium zonatum* was not rejected as the sister group to any lineage of eukaryotes except *Flamella* sp. (formerly Lobosea sp. “Borok” [26]) and apicomplexans (Table S2).

## Discussion

Our findings show that the organisms formerly called protostelids are scattered among *Amoebozoa*. Our trees clearly show: 1) as expected, that protosteloid amoebae are not monophyletic [1], [2], [6], and 2) contrary to predictions, they are not a grade within *Eumycetozoa*, *sensu* Olive [1], [7]. Therefore, our results justify our decision to reject the term protostelids in favor of the strictly descriptive term protosteloid amoebae. While we recognize that the SSU gene presents a problem in resolving deep structure [19], [23], [27], it is ideal for delimiting well supported groups of clearly related organisms.

When we look past the obvious trait of fruiting, we do find that there are five such groups containing only protosteloid amoebae and that their morphological identity is clear when all other detailed stages of the life-cycle are considered. These correspond to Spiegel's groups I, II, III, IV, and Va (see Figure 3 and Table 2 in [2]). In brief these groups are described below. Monographic treatments with formal taxonomic revisions are being prepared separately.

### The Protosteliid Clade - Group I (100% Bootstrap Support)

This group includes the first described protosteloid amoeba, *Protostelium mycophaga* [37]. Its taxa have amoebae with orange pigment and acutely pointed subpseudopodia (Figure 1A, 2A). There are three points of interest within this group: 1) the branch lengths within the species *Protostelium mycophaga* are relatively long, 2) the species which forcibly discharge their spores, *Protostelium nocturnum* [38] and *Protostelium okumukumu* [39], branch basally, and 3) one member of this clade *Planoprotostelium aurantium* makes an amoeboflagellate cell [40]. It was supposed that *Planoprotostelium* was sister to *Protostelium* because of its ability to make flagella and that this ability was lost once, ancestrally to other members of the clade [1], [2], [38], [40]–[42]. Our optimal trees do not support that hypothesis because the genus *Planoprotostelium* is embedded within the protosteliid clade (Figure 3). However, when *Planoprotostelium* was constrained outside of *Protostelium* it branched as sister to *Protostelium*, and this relationship was not rejected by the AU test (Table 2). While the AU test does not let us reject *P. aurantium* as sister to the rest of the protosteliid clade, the similarity of its fruiting body [40] to that of *Protostelium mycophaga* [37] compared to *Protostelium okumukumu* [39] and *Protostelium nocturnum* [38] is quite clear, and we predict that further analysis of a broader taxon sampling of the protosteliid clade will further support *its position within the group rather than as a basal lineage*.

### The Schizoplasmodiid Clade - Group II (100% Bootstrap Support)

The first schizoplasmodiids were described together under the genus name *Schizoplasmodium* [43] based on the plasmodial trophic state that gives rise to the fruiting bodies, and their shared characteristic of a stalk-spore junction with an annular hilum on the spore that articulates with a knob-like apophysis on the stalk (Figure 1B, C, 2B) [2], [43]–[47]. The plasmodial amoeba has both filose and anastomosing subpseudopodia (Figure 2B), and similar “bead on a string” plasmodial mitosis [2], [6], [43]–[46], [48], [49]. Subsequently, schizoplasmodiids were divided into three genera based on variations in fruiting-body stalk length, presence or absence of forcible spore discharge, and presence of an amoeboflagellate in the life-cycle of *Ceratiomyxella tahitiensis* [2], [45], [50]. While our 129 taxa tree groups all three species, *C. tahitiensis*, *Nematostelium ovatum*, and *Schizoplasmodium cavostelioides* together with 100% bootstrap support (Figure 3), a more inclusive mask was required in order to recover the branching order among the three species (Figure 4C). The fine-scale analysis resolved that the two non-flagellates, *N. ovatum* and *S. cavostelioides*, are sister to one another with 96% bootstrap support and a Bayesian posterior probability of 0.95 (Figure 4C), which suggests that the flagellate state may have been lost once in this group as previously supposed [46]. However more taxon sampling of the non-flagellate schizoplasmodiid species, including *Schizoplasmodium obovatum*, *Schizoplasmodium seychellarum*, and *Nematostelium gracile*, will be necessary to resolve this group completely. Given the large number of morphological synapomorphies and the nearly identical SSU rRNA gene sequences, maintaining three genera within the schizoplasmodiid clade may not be well justified. For instance, the separate genus names may well have served to confuse researchers with little firsthand knowledge of protosteloid amoebae, leading them to misclassify members of this clade [51].

### The Soliformoviid Clade - Group III (100% Bootstrap Support)

The genus *Soliformovum* includes two species with identical fan-shaped amoebae with acutely pointed subpseudopodia and indistinct, diffuse nucleoli (Figure 2C) [2], [52], [53]. Both species make a characteristic prespore cell that resembles a “sunny-side-up” fried egg, the character for which the genus is named [2], [52].

### The Cavosteliid Clade - Group IV (65/69% Bootstrap Support)

This is by far the most morphologically diverse clade of protosteloid amoebae. The cavosteliids all have relatively thin amoebae (Figure 2D,E), with filose subpseudopodia, although flagellates and plasmodia also occur as additional stages within some species of the group (see Figure 3 of [2]). Most have round, centrally located nucleoli, except *Schizoplasmodiopsis amoeboidea*, which has indistinct, diffuse nucleoli similar to those seen in *Soliformovum* [2], [52], [54]. They all have sculpturing on their spore walls, and the spores are not deciduous [54]–[57]. Many of these species are common. We were surprised that *Tychosporium acutostipes* branched within this group. Spiegel *et al*. [57] had placed *Tychosporium* as a basal *Protostelium* noting similar prespore cells and some aspects of amoebal morphology, but *Tychosporium* lacked the orange pigment [57]. The cavosteliids are a highly diverse and fascinating group that requires more work. For instance, this clade has the lowest bootstrap support of any of the morphological groups, but support for the group and for nearly every node within the group jumps to nearly 100% if *Cavostelium apophysatum* is removed from the analysis (data not shown). Removal of *Cavostelium apophysatum* from the group was not rejected by the AU test (Table 1). However, we still tentatively accept this clade because of its morphological identity [2].

### The Protosporangiid Clade - Group Va in part (100% Bootstrap Support)

The two species of Group Va that we included, *Protosporangium articulatum* and *Clastostelium recurvatum*, have essentially identical life cycles, essentially identical amoeboflagellates (Figure 2F) and non-flagellated amoebae, and fruiting bodies with 2–4 spores (Figure 1I, J) (see [2], [6], [58]–[62]). Spiegel's [2] group Va also includes *Ceratiomyxa*, for which we do not have sequence data; it has a similar life-cycle and amoeboflagellates [6], [63]. Group Va was thought to be sister to the myxogastrids (Spiegel's group Vb) [2], [6], [61] on the basis of amoeboflagellate ultrastructure. Although the AU test does not allow us to reject this relationship to myxogastrids, we prefer to be skeptical about this hypothesis until further work either supports or fully rejects it. For the same reason, we also remain skeptical about the sister group relationship with *Protostelium* that we recovered with low support in our highest likelihood tree (Figure 3).

### Groups VI & VII

Spiegel suggested that *Endostelium zonatum* and *Protosteliopsis fimicola* might be members of amoeboid groups unrelated to other protosteloid amoebae [2]. Our results support this hypothesis. The placement of *Endostelium zonatum* in the SSU tree is equivocal since it has no strong affinities towards any particular amoebozoan taxon (Figure 3, Table S2) [64]–[66]. Based on similar amoebal morphology and fruiting body development, we thought that our new protosteloid amoeba, isolate LHI05, and *E. zonatum* might be specifically related. However, *E. zonatum* and isolate LHI05 show no close relationship in our highest likelihood trees, though a possible sister group relationship of LHI05 and *E. zonatum*, embedded within the acanthamoebids was not rejected by the AU test (Table 1). If *E. zonatum* were closely related to LHI05, then it would be an acanthamoebid according to the maximum likelihood constrained tree (data not shown). LHI05 is clearly closely related to *Protacanthamoeba bohemica* among the acanthamoebids (Figures 3, 4B) [32]. In fact constraining LHI05 away from *Protoacanthamoeba bohemica* results in a maximum likelihood tree that is strongly rejected by the AU test (Table 1). Two of us, L.L. Shadwick and F.W. Spiegel are currently in the process of describing this new species. *Protosteliopsis fimicola* was originally described as a *Protostelium*, then moved to the monotypic genus *Protosteliopsis* because it was so different (lacking both orange pigment and filose subpseudopodia) from the other species of *Protostelium* [67], [68]. *Protosteliopsis fimicola* robustly groups within the vannellids (Figure 3). In fact, *P. fimicola* displaces *Vannella epipetala* as the sister to *Vannella placida* (Figure 4A) [69]. In addition, *P. fimicola* shares similar patterns of SSU sequence microheterogeneity with both *V. placida* and *V. epipetala* [36], [69]. These molecular phylogenetic findings are consistent with published light and electron-microscopy images [36], [67]–[72]. For instance, *Protosteliopsis fimicola* has the typically conspicuous contractile vacuole, the anterior hyaline veil, the floating form, and the complete lack of uropodia – all typical characters of vannellids [36], [67], [70]. It is clear from Olive's drawings that he was sometimes observing an amoebal state intermediate between the locomotive form and the floating form, which partially explains his constant assertion that *Protosteliopsis fimicola* makes filose pseudopodia, which both *P. fimicola* and vannellids lack [36], [67], [72]. In light of the description of *P. fimicola* and the recent clarification of the genus *Vannella*, it is astounding that no one recognized that *P. fimicola* was a vannellid prior to this study. The misclassification of *P. fimicola* is a testament to both the bias that fruiting induces in the minds of researchers, and the lack of clear, published morphological guidelines for classification of vannellids at the time *P. fimicola* was described [36], [71]–[73].

### Eumycetozoa Question

Protosteloid amoebae are at the crux of the *Eumycetozoa* hypothesis *sensu* Olive [1]. Stalked fruiting body formation was thought to be a synapomorphy of *Eumycetozoa* [1], [7], [8]. Purported eumycetozoans, such as sessile myxogastrids, whose fruiting bodies lacked a stalk were thought to be derived from a stalked ancestor [7]. Other morphological characters were used to support the monophyly of *Eumycetozoa*, *e.g.*, morphology of stalk-producing cells, amoebal morphology and ultrastructure of amoeboflagellates [6], [8], [52], [61], [63], [74], [75]. These characters were also used to delineate the major groups of protosteloid amoebae as discussed above [2]. However, these characters were not considered in the absence of fruiting for comparison to other morphologically similar nonfruiting amoebae; in fact no amoeboid outgroups were considered at all [6], [8], [74], [75]. These additional characters were used, instead, to support fruiting as a character. Just as morphological characters were used to support stalked fruiting as a character, so were early molecular markers [8]–[10]. Here, we have shown with the most extensive taxon sampling that we are aware of for both fruiting and nonfruiting amoebae that: 1) protosteloid ameobae are not monophyletic, and 2) amoebozoans with stalked fruiting are not monophyletic. We have recovered the same well supported clades that others have recovered for myxogastrids [33], dictyostelids [34], and most well to moderately supported clades of nonfruiting amoebae found consistently in the literature [16], [17], [21], [23], [26], [27], [31], [32], [35], [36]. However, support for the deeper relationships among these groups is lacking as in all SSU trees of *Amoebozoa* [16], [17], [19], [21]–[23], [25]–[27], [31], [35], [36], [51], [76].

Rigor demands that we reject the insidious lure of the fruiting body. Our results show, that stalked fruiting, *s.l*., by amoebozoans, though taken as significant, has no *a priori* phylogenetic significance [1], [7], [8]. Rather, it is our view that fruiting has to be taken in context with all the characters of an organism's morphological traits and life history before its significance can be understood.

If we wished to argue for a clade to call *Eumycetozoa* that includes myxogastrids, dictyostelids, and some or all of the protosteliid, schizoplasmodiid, soliformoviid, cavosteliid, and protosporangiid clades, then there are several nodes we could select on the tree as basal to such a group (Figure 3). We think this is unwise for two reasons. First, while it is interesting that our trees show that there may be protosteloid sister groups to both dictyostelids and myxogastrids, the affinities are very poorly supported. Second, it must be recognized that almost all of the groups in the part of the tree that includes most of the fruiting organisms have extremely long branches; thus, some of the deep structure in our tree could simply be a result of long branch attraction [76]. We have attempted to alleviate some long branch effects by using a conservative inclusion set of unambiguously alignable sequence and by including multiple representatives of each lineage where possible, but we cannot confidently rule out long branch attraction. Therefore, we think formal taxonomic revision should be restricted only to well supported clades. Further work, such as comparative genomics, will be necessary to resolve the uncertain deeper relationships. We are inclined to be very conservative when using our results to revise the higher level taxonomy of these organisms. In fact, we are strongly disinclined to even propose informal names for poorly supported groups that happen to occur in our highest likelihood tree unless subsequent research provides more support for them. Taxonomic revisions based on poorly resolved phylogenetic nodes only clutter the literature with names that can lead to confusion. Therefore we think it is best, for now, to relinquish the concept of *Eumycetozoa*.

We would like to make our point as strongly as possible. Our results show that stalked fruiting is widespread among the *Amoebozoa*. Thus, *if* we were presumptuous enough to accept 1) that our tree of this amoeboid supergroup (Figure 3) is true, 2) that stalked fruiting in the supergroup has only one origin, and 3) that fruiting is important as a defining character, then the name *Eumycetozoa* Zopf 1885 would be correct for the whole supergroup since the name has taxonomic priority over the name *Amoebozoa* Lühe, 1913 [1], [77]–[79]. However, at least two issues need to be resolved before we would consider formally renaming *Amoebozoa*. First, the evolutionary and developmental origin of fruiting must be understood. Second, the higher order relationships among fruiting and nonfruiting lineages must be resolved.

## Materials and Methods

### Cultures

At least one protosteloid amoeba from each major morphological group of Spiegel [2] was sampled. Type cultures were used where available, as were multiple isolates. When necessary we isolated organisms from nature into monoeukaryotic or dieukaryotic culture as previously described [2], [3], [21] and other cultures were acquired from culture collections (Table 3).

**Table 3:** Protosteloid Amoeba Cultures Used.

Name	Collection #	Source	Culture Collection	Food	Group	Genbank
*Cavostelium apophysatum* ^t^	G-17	supplied	ATCC 38567	Fla	IV	FJ766476
*Ceratiomyxella tahitiensis*	HIO4-93L-1	collected	UA	M,*Cl*	II	FJ544419
*Clastostelium recurvatum*	NZ05-10a-4	collected	ATCC PRA-189, UA	*Kp*	Va	FJ766474
*Endostelium zonatum*	LHIO5M6a-1	collected	ATCC PRA-191, UA	F	VII	FJ766469
*Nematostelium ovatum*	JDS 6241	collected	UA	M,K	II	FJ544420
*Protosporangium articulatum*	1-Bg3-9-1	N/A	N/A	U	Va	FJ792705
*Protostelium mycophaga ^t^*	Type	collected	ATCC PRA-154, UA	*Rm*	I	FJ766484
*Protostelium mycophaga*	HI04 85a-1b	collected	UA	*Rm*	I	FJ766483
*Protostelium nocturnum*	LHI05M6a-1a	collected	ATCC PRA-194, UA	Fla	I	FJ766481
*Protostelium okumukumu ^t^*	HIO4-37a-1a	collected	ATCC PRA-156, UA	*Rm*	I	FJ766482
*Protosteliopsis fimicola*	H76-34	purchased	CCAP 1569/I,	F	VI	FJ766470
*Protosteliopsis fimicola*	H76-34	purchased	CCAP 1569/I,	F	VI	FJ766471
*Protosteliopsis fimicola*	Ken-A ‘20DE’	collected	UA	F	VI	FJ766472
*Protosteliopsis fimicola*	OM05-6218-1	collected	UA	F	VI	FJ766473
*Schizoplasmodiopsis*						
* pseudoendospora*	PBR-G5-1	collected	ATCC PRA-195	Fla	IV	FJ766475
*Schizoplasmodiopsis amoeboidea ^t^*	RA81-20	supplied	ATCC 46943	Fla	IV	FJ766477
*Schizoplasmodiopsis amoeboidea*	BG7A-12B	collected	UA	Fla	IV	FJ766478
*Schizoplasmodium cavosteliodes*	NZ05-24L-2	collected	ATCC PRA-197, UA	M,K	II	FJ544418
*Soliformovum expulsum ^t^*	YAP 76-9	supplied	ATCC 48083	M	III	FJ766479
*Soliformovum irregularis ^t^*	Mex 61-81	supplied	ATCC 26826	*Ec*	III	FJ766480
*Tychosporium acutostipes*	NZ05-15a-2	collected	ATCC PRA-196, UA	Fla	IV	FJ792704
*Tychosporium acutostipes*	KEA-11A-L	collected	UA	Fla	IV	FJ792703
Unnamed	LHI05M5g-1	collected	ATCC PRA-198, UA	F	none	FJ792702
Unnamed	LHI05M5g-1	collected	ATCC PRA-198, UA	F	none	FJ794612

Name abbreviations: *^t^* = Type cultures. Collection number = collection number of the source material used to isolate the culture/collection. Source abbreviations: supplied = supplied by, collected = isolated from substrates collected in the field, purchased = Purchased from culture collection. Culture Collection abbreviations: ATCC = American Type Culture Collection (Eumycetozoan Special Collection), UA = University of Arkansas (cryopreserved), CCAP = Culture Collection of Algae and Protozoa. Food organism abbreviations: Fla20 = *Serratia liquefaciens* strain Florida 20 of Olive ATCC BAA-1466, M = *Dyadobacter* sp. strain Malaya (MAL 82 of Olive) ATCC BAA-1468, K = *Tilletiopsis* sp. strain Kitani of Olive, *Ec* = *Escherichia coli* ATCC 23432, *Cl* = *Cryptococcus laurentii* (kindly provided by E.F. Haskins), *Rm* = *Rhodotorula mucilaginosa* of Olive ATCC 14023, *Kp* = *Klebsiella pneumonia* ATCC 23432, F = *Sphingomonas sp.* Strain FLAVO ATCC BAA-1467, U = uncultured. Genbank = genbank accession number.

Protosteloid amoebae were grown on weak malt yeast extract agar plates (wMY) (0.002 g malt extract, 0.002 g yeast extract, 0.75 g K_2_HP0_4_, 15.0 g Difco Bacto Agar, 1.0L deionized [DI] H_2_0) with appropriate food organisms (Table 3) in the laboratory at ambient temperatures (approx 21–25°C) [2], [3]. *Protosporangium articulatum* could not be established in culture.

All cultures were vouchered by rigorous microscopical examinination on a Zeiss Axioskop 2 Plus under the 10×dry differential interference contrast (DIC) and bright field (BF), 40×dry (DIC/Phase contrast (PC)), and 63×oil (DIC) to verify the proper taxon identification of each organism, and to check for possible contaminants. All cultured organisms were observed to form fruiting bodies in culture. All cultures were digitally photographed using AutoMontage (Syncroscopy, Frederick, MD). All cultures, even those obtained from ATCC and CCAP except *Schizoplasmodiopsis pseudoendospora* (which later succumbed to a bacterial contamination) were put into a viable frozen stasis in liquid nitrogen and are stored at the University of Arkansas. Many were also depostited at the ATCC (Table 3).

### DNA extraction

DNA from cultures was made available for PCR by using a chelex method that requires little starting material and provides few chances for cross contamination of reagents [80]. Chelex solution was 6% (w/v) chelex100 resin (Bio-Rad Laboratories, Hercules, CA) in double distilled, diethylpyrocarbonate treated H_2_0. Under a dissecting microscope (Nikon SMZ 1500), organisms were scraped off their media using an ethanol-flamed spear-point needle and placed into 150 µl of chelex solution. Negative controls include 1) chelex solution only 2) chelex solution + food organism. All were placed into a thermal cycler for 4 hours at 56°C followed by 30 min. at 98°C, then stored at −20°C until needed. *Protosporangium articulatum* was treated differently because we were not able to grow it in culture. Fruiting bodies on their natural substrate were vouchered through photomicroscopy (Figure 1J), then approximately 30 spores were collected with an insect pin (see [21]). DNA was extracted from the collected spores using the MasterPure Complete DNA and RNA Purification Kit (Epicentre, Madison, WI) according to manufacturer's protocols.

Two additional sequences were included to increase phylogenetic signal from the *Myxogastria. Lindbladia tubulina* fruiting bodies were a kind gift from Sergey Karpov, Herzen State Pedagogical University, St. Petersburg, Russia. *Trichia decipiens* fruiting bodies were collected from their natural substrate in Halifax Nova Scotia, Canada during the summer of 2001. DNA from each myxogastrid was isolated from spores or maturing plasmodium using PureGene tissue lysis kit, per manufacturer's recommendations.

### PCR and DNA sequencing

The SSU from protosteloid amoebae were amplified with either “universal” eukaryote SSU primer pairs Medlin A, Medlin B [81], 30F, 1492R, 5′SSU17!, or specifically designed biased primers PmycF1, PmycR2, Myxo3′ (Table 4). The SSU of *Protosporangium articulatum* was obtained by a nested PCR protocol using Medlin A : B in the first PCR reaction that served as template for a second PCR using 30F : 1492R. For the two myxogastrids, *L. tubulina* and *T. decipiens,* SSU rDNA was amplified with the “universal” eukaryotic primers Medlin A : B [81], cloned and sequenced as previously described [21].

**Table 4:** SSU rDNA primers for protosteloid amoebae.

Medlin A : B	Medlin A : PmycR2	PmycF1 : PmycR2	30F : 1492R	5′SSU17! : Myxo3′
*S. irregularis*	*T. acutostipes* NZ	*P. mycophaga*	*P. articulatum* 2°	*S. pseudoendospora*
*C. recurvatum*		*P. nocturnum*		*P. fimicola*
*E. zonatum*		*S. amoeboidea*		
*S. cavosteliodes*		*N. ovatum*		
*C. apophysatum*		*C. tahitiensis*		
*T. acutostipes* KE		*S. expulsum*		
*P. articulatum* 1°		isolate LHI05		

PmycF1: 5′ TCC TGC CAG TAG TCA TAT GCT 3′ , PmycR2: 5′ GCA GGT TCA CCT AGG GAG 3′, Medlin A: 5′ CCG AAT TCG TCG ACA ACC TGG TTG ATC CTG CCA GT 3′, Medlin B: 5′ CCC GGG ATC CAA GCT TGA TCC TTC TGC AGG TTC ACC 3′ [81], 30F: 5′ AAA GAT TAA GCC ATG CAT G 3′, 1492R: 5′ ACC TTG TTA CGA CTT 3′, 5′SSU17!: 5′ CTG GTT GAT CCT GCC AG 3′, and Myxo 3′:5′ TAA TGA TCC AAA GGC AGG TTC ACC TAC 3′.

For the SSU from protosteloid species, a stepdown thermal cycling program was used with Platinum Blue RTS PCR Super Mix (Invitrogen, Carlsbad, CA) in 20 µl reactions with the following cycling parameters: preheat lid 105°C, initial denaturation 94°C 1 min., followed by 5 cycles of 94°C 30 sec., 1 min. for primer annealing at 60°C followed by 3 min. 5 sec. elongation at 72°C. Then 9 cycles were done with denaturation 94°C for 30 sec., with an initial annealing temperature of 59°C that decreased 1°C/cycle to a final 50°C annealing, elongation for 3 min. 5 sec. at 72°C, followed by 20 cycles as above with a 50°C annealing temperature and an elongation time of 2 min. 5 sec. PCR products were either sequenced directly after removal of unincorporated nucleotides and primers using QIAquick Gel-Extraction kit (Qiagen, Valencia, CA), or they were T/A cloned into TOPO vector pCR4 and transformed into TOP 10 *Escherichia coli* cells per manufacturer's instruction (Invitrogen). For cultures grown on yeast (where no size difference between the SSU of the yeast and amoeba was seen), amplified and cloned SSU inserts were PCR amplified directly from transformed bacterial colonies and screened by TaqI restriction fragment length polymorphism with TaqαI restriction endonuclease (New England Biolabs, Ipswich, MA) to distinguish between yeast SSU clones and protosteloid SSU clones.

DNA sequencing reactions were performed using big-dye chemistry and resolved on an Applied Biosystems 3100 Genetic Analyzer (Foster City, CA). PCR products were sequenced directly, where possible in both orientations. Otherwise, one or two clones and/or a pool of 6–10 clones were sequenced fully in both orientations. Partial sequence was often obtained from additional clones.

For protosteloid amoebae that grew with yeast as food organisms, we partially sequenced one fungal clone (identified by restriction fragment length polymorphism), and fully sequenced all available (1 to 10) protosteloid clones. In several cases, within-isolate SSU sequence microheterogeneity was observed. In these cases pooled clones and/or PCR products were sequenced plus one or two individual clones through regions of heterogeneity (*Protosteliopsis fimicola*, *Endostelium zonatum*, isolate LHI05). In cases of within-isolate microheterogeneity that inhibited sequencing through certain regions of pooled clones/PCR products, all individual cloned SSUs were sequenced and included in preliminary trees. In all cases those partial sequences clustered tightly together with other sequences from the same isolate. An individual clone from each of these isolates was used for subsequent analyses. All new sequences were accessioned in GenBank (Table S1).

### Phylogenetic analyses

Protosteloid amoeba SSU sequences were hand aligned into an existing SSU rDNA multiple sequence alignment in MacClade (Sinauer Associates, Sunderland, MA) [82]. Ambiguous regions in the alignment were excluded from phylogenetic analyses. The largest data set consisted of 129 taxa that span the known diversity of *Amoebozoa* plus a wide array of outgroup taxa that included at least two members from most other major eukaryotic lineages.

Multiple representatives from each sequenced amoebozoan lineage were included to deeply sample the molecular diversity available within *Amoebozoa* and to assess congruence with other amoebozoan phylogenies. Phylogenies were inferred using maximum likelihood (ML) as implemented in PAUP* 4.0 [83], RAxML 7.0.4 [84], and GARLI 0.96 [85] using a GTR + Γ + I model of nucleotide substitution (except RAxML which implemented a GTR CAT model for topology search and a GTR + Γ model for tree optimization, both with 20 rate categories one of which essentially corresponds to invariant sites). Specific model parameters were determined using ModelTest 3.7 [86] selected using the Akaike information criterion (AIC) [87] for analyses run in PAUP*, while RAxML and GARLI were allowed to estimate models during their respective analyses. A single optimum ML tree was inferred in PAUP* while the highest likelihood tree was identified from 300 RAxML and 300 GARLI runs each starting from a different parsimony tree. The optimum ML tree was inferred in RaxML with branch lengths optimized in PAUP* is shown in Figure 3. Topological support for branches was assessed from the consensus of 1,000 ML bootstrap trees inferred in RAxML and GARLI. For the 129 taxa data set Bayesian parameters failed to converge even after 30 million generations.

For all other finer-scale phylogenetic analyses, both ML and Bayesian analyses were performed. ML analyses were performed in PAUP* with the nucleotide substitution model and specific parameters selected for each dataset using ModelTest as implemented in PAUP* [86]. For Bayesian analyses we used Mr.Bayes 3.1.2 [88] with 4 Markov chain Monte Carlo (MCMC) chains in each of two independent runs with nst = 6 and rates set to invgamma (corresponding to a GTR + Γ +I model of nucleotide substitution). Trees generated prior to convergence of parameters were discarded as ‘burn-in’. Convergence was detected only after the standard deviation of split frequencies dropped below 0.01 and the sump function provided in Mr.Bayes and the program Tracer (part of the BEAST package) [89] all indicated convergence.

### Hypotheses testing

Phylogenetic tree topologies conforming to a variety of specific hypotheses were tested in a likelihood framework. Hypothesis testing was done on the 129 taxa tree (Figure 3) using the Approximately Unbiased (AU) test in the program Consel 0.1i [90], [91]. Consel compares likelihoods, but the likelihoods calculated by the programs RAxML and GARLI, which were used to generate topologies and estimate models, are not directly comparable [84], [85]. Also, each topology generated by RAxML and GARLI has unique model parameters estimated with it. A single set of model parameters was needed for input into PAUP* so that comparable likelihood values could be calculated for every topology no matter how it was generated. RAxML and GARLI each produce a highest likelihood topology (as calculated within the program). We considered the model parameters associated with that highest likelihood topology to be the optimal model parameters produced by that program. Thus PAUP* was used to calculate likelihoods using the optimal model parameters estimated by RAxML for all of the tree topologies generated in RAxML and GARLI. Then PAUP* was used to calculate the same likelihoods using the optimal model parameters estimated by GARLI. The likelihoods of tree topologies produced by the two methods produced distributions that overlapped almost entirely. Paup* was used to calculate likelihoods for all topologies using the model estimated by RAxML for all subsequent analyses. Six hundred ten constraint topologies were created by manually constraining specific taxa to branch together followed by reoptimization of the branching among the remaining taxa. Reoptimization was performed by inferring three ML trees in RAxML with a GTR + Γ model (as specified in RAxML) and keeping the highest likelihood of these trees. Thus, 610 constraint tree topologies (hypotheses) were generated. For a list of constraints see table S2. Site likelihoods, required for import into Consel, were calculated from the optimal ML topologies, the 610 specific constraint topologies and a set of plausible topologies consisting of the 1,000 RAxML and 1,000 GARLI bootstrap topologies in PAUP* using the RAxML substitution model. Significant differences in the likelihood among all trees were tested by the AU, Shimodaira-Hasegawa (SH), and Kishino-Hasegawa (KH) tests as implemented in Consel 0.1i [90], [91].

## Supporting Information

Table S1 Genbank Accession Numbers for Additional Sequences Used in Phylogenetic Analyses. Bold font highlights protosteloid species. * = organisms sequenced in this study.
(0.05 MB XLS)

Table S2 Constrained Taxa Abbreviations and Constraints Used for Statistical Tests. Each well supported amoebozoan lineage is constrained with all other amoebozoan and outgroup lineages. The first two columns refer to the taxon and its abbreviation in the constraint. The taxa constrained to branch together are within the parentheses. For more complex constraints, where some taxa are constrained away from a group the notation used is ((taxa constrained together) taxa constrained away);. All other taxa (from 129 taxa dataset see figure 3) that are not constrained in or out of a group are ommitted from this table for ease of reading. Constrained trees (not shown) were built in RAxML and likelihood scores (-lnL) were estimated in PAUP* using the constraints shown. Significant differences in the likelihood among all trees were tested by the AU, SH, KH tests as implemented in Consel [90].
(0.22 MB XLS)
